# Spectral Characteristics of Dissolved Organic Matter and Their Associations with Heavy Metal Distribution in Multi-Media of a Typical Frozen Eutrophic Lake

**DOI:** 10.3390/toxics14060527

**Published:** 2026-06-18

**Authors:** Zhijian Lv, Xuezheng Yu, Weiying Feng, Yu Qiao, Chia Min Ho, Jiayue Gao, Fanhao Song, Wenhuan Yang, Sundaravelpandian Kalaipandian

**Affiliations:** 1 School of Materials Science and Engineering, Beihang University, Beijing 100191, China; 2 State Key Laboratory of Environmental Criteria and Risk Assessment, Chinese Research Academy of Environmental Sciences, Beijing 100012, China; 3 School of Energy and Environment, Inner Mongolia University of Science and Technology, Baotou 014010, China; 4 Queensland Alliance for Agriculture and Food Innovation, The University of Queensland, Brisbane City 4072, Australia

**Keywords:** dissolved organic matter, EEMs-PARAFAC, ice–water–sediment, cold arid region

## Abstract

In cold arid regions, the relationships between dissolved organic matter (DOM) characteristics and heavy metal distributions across ice, water, and sediment interfaces remain insufficiently resolved. This study characterized DOM spectral features and examined their associations with measured metal distributions in a typical frozen eutrophic lake using excitation–emission matrices coupled with parallel factor analysis (EEMs-PARAFAC), ultraviolet-visible absorption spectroscopy (UV-Vis), and Fourier-transform infrared spectroscopy (FTIR). Protein-like substances dominated ice DOM, whereas water and sediment-derived DOM contained more humified fluorescent components. Fluorescence indices confirmed a primarily biological origin across all media, with ice showing the highest autochthonous microbial contribution (BIX = 1.23) but the lowest humification (HIX = 0.26), suggesting a greater contribution of recently produced protein-like fluorescent DOM in the ice samples. Water DOM showed the highest average HIX (1.88), followed by sediment-derived DOM (0.61) and ice DOM (0.26). The measured hydrochemical conditions, including weak alkalinity, elevated total dissolved solids (TDS), and locally low dissolved oxygen, provide environmental context for differences in metal distributions. Exploratory Spearman analysis at 17 matched water stations identified the strongest DOM–metal associations for HIX-As (rho = 0.474, *p* = 0.054) and FI-Zn (rho = 0.471, *p* = 0.056), indicating that DOM optical properties provide testable indicators of metal-distribution patterns but should be combined with direct binding and speciation measurements for mechanistic confirmation. Because ice was collected in January 2021, whereas water and sediment were collected in October 2020, cross-medium differences are interpreted as between-campaign associations rather than synchronous partitioning. These findings provide a basis for targeted winter monitoring and future binding, speciation, and freeze-concentration experiments in shallow eutrophic lakes.

## 1. Introduction

Under the dual pressures of human activities and global climate change, lakes in cold and arid regions are facing severe environmental challenges, including water quality deterioration and ecosystem degradation, which directly threaten aquatic ecological security and regional drinking water safety [1,2]. In China, lake-water quality is evaluated under the Environmental Quality Standards for Surface Water (GB 3838-2002) [3], and eutrophication remains a major environmental concern for Chinese lakes [4].

Dissolved organic matter (DOM) is one of the most active components in lake systems, present in ice, water, and sediment. It plays a vital role in nutrient cycling, pollutant transport, and biogeochemical processes [5]. Due to its high solubility, DOM influences the behavior and fate of organic contaminants, heavy metals, and nutrients, thus affecting their bioavailability, toxicity, and overall environmental impact [6,7]. However, the dynamics of DOM composition and its interaction with environmental factors—especially under microbial regulation—remain inadequately understood [8,9].

In lake ecosystems, ice, water, and sediment each form a unique functional medium. Between them, continuous physical, chemical, and biological processes drive the transfer and transformation of nutrients and pollutants, which in turn affect the concentration and speciation of substances in the overlying water and exacerbate the degree of eutrophication [10]. Under ice cover in winter, light penetration, temperature, and dissolved oxygen levels undergo significant changes, altering the biochemical processes of organic matter such as plant and animal residues [11]. Thus, exploring the migration and transformation of DOM across the ice–water–sediment system is essential to understanding the evolution of eutrophication during the ice-sealed period and can provide new insights into the control of internal pollution in seasonally frozen lakes.

Currently, DOM research focuses primarily on its sources, composition, distribution, structural characteristics, and biogeochemical behavior [12]. The main analytical techniques include three-dimensional excitation–emission matrix spectroscopy (EEMs), Fourier transform infrared spectroscopy (FTIR), and ultraviolet–visible (UV–Vis) spectroscopy, which are powerful tools for characterizing the composition, aromaticity, functional groups, and humification degree of DOM [13,14,15]. Despite this, most studies have been conducted during the ice-free period, and systematic research on the differences in DOM across multiple media—ice, water, and sediment—under long-term ice cover conditions is still lacking.

Lake Ulansuhai, a typical shallow macrophytic lake in the cold arid region of Inner Mongolia, China, has suffered from serious eutrophication over the past two decades. Lake Ulansuhai is located in Urad Front Banner, Bayannur City, Inner Mongolia Autonomous Region (40°36′–41°03′ N, 108°43′–108°57′ E), and its shallow macrophyte-dominated basin, long ice-covered period, and sustained influence of agricultural return flow make it a representative system for studying DOM behavior in cold arid eutrophic lakes. Non-point source pollution from agricultural return flow has led to continuous inputs of nitrogen and phosphorus, resulting in the frequent occurrence of algal blooms and long-term inferior Class V water quality [16,17]. Its shallow basin, macrophyte-dominated habitat, sustained influence of agricultural return flow, and prolonged annual ice-covered period provide a representative setting for examining winter DOM patterns and hydrochemical constraints in an arid lake environment [1,4,14]. The ice-frozen period lasts for 5 to 7 months each year. The ice-sealed period is a key hydrological feature of this lake. During winter, pollutants accumulate in water and sediment, and the growth of ice layers can even concentrate underlying pollutants, posing potential risks to ecosystem health [1,4,5]. Moreover, the ecological impacts of ice cover are often underestimated due to difficulties in winter sampling and the traditional assumption that low temperature ensures environmental stability [7,18,19]. This has resulted in significant gaps in winter lake research compared to studies of summer or ice-free lakes.

Given the above background, there is an urgent need to investigate the composition, sources, and multi-interface behavior of DOM in ice-covered eutrophic lakes, as well as its associations with measured metal distributions across ice, water, and sediment. This study selected Lake Ulansuhai as a typical case and applied integrated spectroscopic techniques to (1) characterize and compare the molecular composition and structural features of DOM in ice, water, and sediment; (2) identify the potential sources and humification degree of DOM in different media; (3) examine measured ion and metal distributions alongside DOM spectral patterns; and (4) distinguish observational associations from mechanisms requiring direct validation. The findings are expected to provide a scientific basis for pollution control and ecological restoration of seasonal ice-covered lakes, while defining the additional measurements required to evaluate DOM-mediated metal behavior under ice.

The study hypothesis was that media-specific DOM spectral signatures and hydrochemical conditions would be associated with differences in measured metal distributions, whereas direct metal–DOM binding mechanisms would require independent validation.

## 2. Materials and Methods

### 2.1. Study Area

Lake Ulansuhai (40°36′–41°03′ N, 108°43′–108°57′ E) is located in Urad Front Banner, Bayannur City, Inner Mongolia Autonomous Region, China. It represents a typical shallow macrophyte-dominated lake in the arid western part of the Inner Mongolia Plateau. Its extensive aquatic vegetation, shallow water column, eutrophic condition, and seasonal ice cover provide an environmentally relevant setting for examining winter DOM characteristics in a cold arid lake system [1,4,14]. As one of the eight largest freshwater lakes in China, Lake Ulansuhai covers a water area of approximately 333.48 km^2^. Among this, emergent plants occupy about 220 km^2^, while submerged vegetation covers roughly 110 km^2^. More than three-quarters of the lake has a water depth ranging between 0.8 and 1.0 m, with an estimated water storage capacity of about 2.5–3 × 10^8^ m^3^ [1,4,14].

### 2.2. Sample Collection and Pretreatment

**Figure 1:**
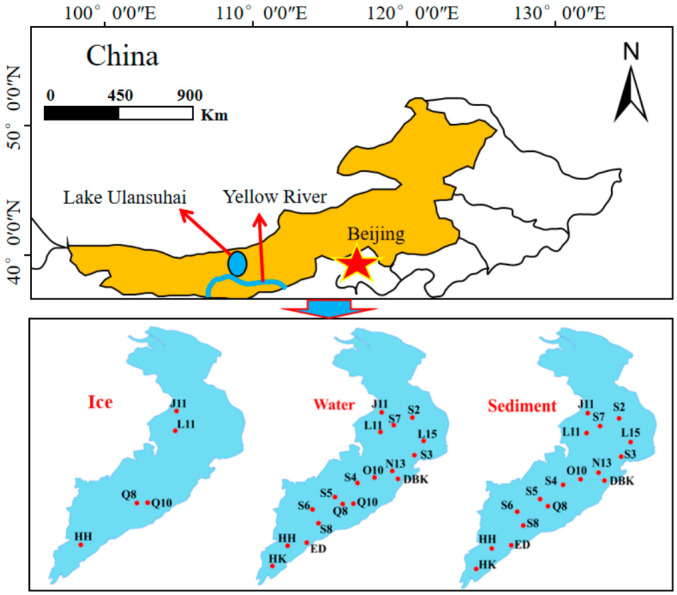
Location of Lake Ulansuhai in China, main inflowing rivers, and distribution map of sampling points in lake ice, water, and sediment.

As shown in Figure 1, a total of 147 samples were collected: water and sediment samples were each taken from 17 sites from Lake Ulansuhai in October 2020 with three replicates per site; ice samples were collected from 5 sites, where each site was divided into three layers (upper, middle, and lower), and three replicates were taken from each layer. Based on the distribution of pollution sources and the hydrodynamic characteristics of Ulansuhai Lake, the lake is spatially divided into 2 km × 2 km square grids. Water sampling points are arranged in a plum-blossom pattern at the grid intersections, located using GPS, and the numbering of the sampling points continues the numbering system that the research group has used for over 20 years [20]. The sampling locations for water were: O10, DBK, J11, ED, HH, L11, L15, N13, Q8, Q10, S2, S3, S4, S5, S6, S7, and S8. Sediment samples were collected from: O10, DBK, J11, ED, HH, L11, L15, N13, Q8, HK, S2, S3, S4, S5, S6, S7, and S8. In January 2021, ice samples were collected from five locations (HH, J11, L11, Q8, O10). At each site, ice was sampled from three distinct layers: upper (U), middle (M), and lower (D). This sampling strategy resulted in 15 ice samples, labeled as follows: HHU, HHM, HHD, J11U, J11M, J11D, L11U, L11M, L11D, Q8U, Q8M, Q8D, O10U, O10M, and O10D.

Water samples were collected from the mid-depth water column (approximately 0.5 m below the water surface) using pre-cleaned polyethylene samplers. Surface sediment samples were collected with a stainless-steel grab sampler, and the upper 0–10 cm sediment layer was retained after removing visible plant residues and shell fragments. Ice samples were collected using a stainless-steel ice drill/corer; the total ice thickness was measured in situ at each site, and each complete ice core was immediately divided into three equal sections representing the upper, middle, and lower layers. All samples were stored in acid-washed polyethylene containers, transported to the laboratory in insulated coolers, and processed as soon as possible.

### 2.3. Analysis of Basic Water Parameters and DOM Extraction

Field measurements: Key water quality parameters, including pH, dissolved oxygen (DO), and total dissolved solids (TDS), were measured in situ at the time of sampling using a portable multiparameter water-quality meter (Hach HQd, Hach Company, Loveland, CO, USA). Laboratory analyses: Sediment samples were freeze-dried for 48 h until completely dry, followed by removal of impurities, grinding, and sieving through a 200-mesh screen for subsequent analysis. Fourier-transform infrared (FTIR) spectroscopy was applied to characterize the functional group information of DOM from sediment using a [Nicolet iS50] (Thermo Fisher Scientific, Waltham, MA, USA). For ice, water and sediment samples, ultraviolet-visible (UV-Vis) spectroscopy was performed with a [Hitachi U-3900] (Hitachi High-Technologies Corporation, Tokyo, Japan), and fluorescence spectroscopy was conducted with a [Hitachi F-7000] (Hitachi High-Technologies Corporation, Tokyo, Japan).

To extract DOM from sediments, 0.5 g of each processed sediment sample was mixed with 25 mL of deionized water in a 50 mL centrifuge tube. The mixture was shaken at 250 rpm for 16 h at 22 °C, then centrifuged at 4000 rpm for 20 min at 4 °C. The supernatant was filtered through a 0.45 μm membrane and transferred into a new 50 mL centrifuge tube for subsequent UV-Vis and fluorescence measurements. All aqueous solutions used in the experiments were prepared using Milli-Q ultrapure water (Millipore, 18.2 MΩ·cm). In this manuscript, the term “sediment extract” denotes this operationally defined water-extractable fraction and does not denote in situ sediment pore water. Dissolved organic matter (DOM) is a class of organic compounds rich in conjugated systems. In aqueous solutions, their ultraviolet (UV) absorption spectra exhibit strong signals. The absorption coefficients (a_254_, a_365_, a_436_, a_465_, a_665_) can be used to characterize the UV spectral features, with the calculation method given by Equation (1).

α_λ_[m^−1^] = 2.303 ∙ Abs_λ_/L (1)

In this equation, L is the cuvette path length (commonly 0.01 m), λ is the wavelength, and Abs is the absorbance. The absorbance index ratios E_2_/E_3_, E_2_/E_4_, and E_4_/E_6_ are defined as a_254_/a_365_, a_254_/a_436_, and a_465_/a_665_, respectively. These ratios can indicate the degree of humification, the source of organic matter, and the aggregation degree and conjugation degree of organic matter forming ring structures through double or triple bonds.

### 2.4. Three-Dimensional Excitation–Emission Matrix (EEM) Spectroscopy

Fluorescence spectra were measured using a Hitachi F-7000 fluorescence spectrophotometer (Hitachi High-Tech Corporation, Tokyo, Japan). The excitation (Ex) wavelength was scanned from 200 to 600 nm with a bandwidth of 5 nm, and the emission (Em) wavelength was recorded from 250 to 600 nm, also with a 5 nm bandwidth. The scanning speed was set at 600 nm·min^−1^. The resulting EEM datasets were analyzed using parallel factor analysis (PARAFAC) in MATLAB (R2018a) with the DOMFluor toolbox. The model was validated through split-half analysis and inspection of the core consistency to determine the optimal number of fluorescent components [1,9,20].

Several spectral indices were calculated to characterize the DOM properties: (1) Fluorescence Index (FI): Defined as the ratio of emission intensity at 450 nm to that at 500 nm, obtained at an excitation wavelength of 370 nm. This index is used to distinguish between terrestrial and microbial sources of DOM [21]. (2) Humification Index (HIX): Calculated as the area under the emission spectrum from 435 to 480 nm divided by the area from 300 to 345 nm, at an excitation of 254 nm. HIX reflects the degree of humification of DOM. (3) Biological Index (BIX): Determined as the ratio of emission intensity at 380 nm to that at 430 nm at an excitation of 310 nm, indicating the proportion of recently produced autochthonous DOM. (4) Absorption indices: The ratios E2/E3 (a_254_/a_365_), E2/E4 (a_254_/a_436_), and E4/E6 (a_465_/a_665_) were calculated from the UV-Vis absorption data. E2/E3 is inversely related to molecular weight, E2/E4 is associated with DOM source, and E4/E6 reflects the degree of condensation and conjugation in humic substances.

### 2.5. Fourier-Transform Infrared (FTIR) Spectroscopy Analysis

The molecular composition and functional groups of dissolved organic matter (DOM) extracted from sediment samples were characterized using Fourier-Transform Infrared spectroscopy (FTIR). Spectra were collected on a Thermo Scientific Nicolet iS50 FTIR spectrometer equipped with a deuterated triglycine sulfate (DTGS) detector. Each freeze-dried and homogenized sediment DOM sample was mixed with spectroscopic-grade potassium bromide (KBr) at a ratio of 1:100 and pressed into a transparent pellet under hydraulic pressure. Background scans were collected prior to sample analysis to correct for atmospheric interference.

Spectra were acquired over a wavenumber range of 4000–400 cm^−1^, with a resolution of 4 cm^−1^ and 64 accumulated scans per sample to ensure high signal-to-noise ratio. The resulting spectra were processed using OMNIC™ software (Version 9.0, Thermo Fisher Scientific, Waltham, MA, USA), which included baseline correction, atmospheric suppression, and smoothing. Characteristic absorption peaks were identified and assigned to specific functional groups based on established literature: 3400 cm^−1^ (O-H stretching, alcohols/phenols), 2920 and 2850 cm^−1^ (C-H stretching, aliphatic groups), 1650 cm^−1^ (C=O stretching, amides/quinones), 1550 cm^−1^ (N-H bending, amides II), 1400 cm^−1^ (C-O-H bending, carboxylic groups), and 1050 cm^−1^ (C-O stretching, polysaccharides). This analysis provided insight into the structural properties and major functional groups present in the sediment-derived DOM, complementing the fluorescence and UV-Vis spectroscopic data. Because FTIR was performed only for sediment-derived DOM, FTIR-based structural interpretations are restricted to sediment extracts and are not extrapolated to ice or water DOM. FTIR analysis was focused on sediment-derived DOM because sediment extracts were expected to contain more structurally complex water-extractable organic matter and provided sufficient material for KBr-pellet preparation; ice and water DOM were characterized by EEM-PARAFAC and UV-Vis in this study, while future work should extend FTIR or other molecular-level analyses to concentrated DOM isolates from all three matrices.

### 2.6. Analysis of Major Ions and Heavy Metals

Concentrations of major anions (F^−^, Cl^−^, Br^−^, NO_3_^−^, SO_4_^2−^) and cations (Na^+^, NH_4_^+^, K^+^) in water, ice, and sediment extracts were determined using ion chromatography (IC, Thermo Scientific™ Dionex™ ICS-1100, Thermo Fisher Scientific, Sunnyvale, CA, USA). For anion analysis, a Dionex IonPac AS23 analytical column (4 × 250 mm) was employed with a carbonate/bicarbonate eluent under a flow rate of 0.60 mL/min. Cation separation was carried out using a Dionex IonPac CS12A column (4 × 250 mm) with methanesulfonic acid as the eluent at a flow rate of 0.10 mL/min. The column temperature was maintained at 40 °C for both analyses, and the injection volume was 20 μL.

Metals and metalloids (Cu, Fe, Mn, Cd, Zn, Ni, Cr, Hg, As, Pb, Se, Sb, and Ba), together with the major elements Ca and Mg, in water samples were quantified using inductively coupled plasma mass spectrometry (ICP-MS, Agilent 7900, Agilent Technologies, Santa Clara, CA, USA). Calibration standards were prepared from certified multi-element solutions, and internal standards were used to correct for matrix effects and instrument drift. The RF power was set to 1550 W, and the plasma gas flow was maintained at 15 L/min. Each sample was measured in triplicate to ensure analytical precision. Quality assurance was performed by analyzing NIST SRM 1643f (Trace Elements in Water), procedural blanks, and duplicate samples with each batch of samples, achieving recovery rates of 92–108% for all reported elements. All reagents used were of analytical grade, and ultrapure water (Milli-Q, 18.2 MΩ·cm) was used throughout the sample preparation and dilution processes. The analyzed elements were selected to cover three categories: (i) priority toxic metals and metalloids of regulatory concern in surface-water assessment (As, Cd, Cr, Cu, Pb, Hg, Se, and Zn); (ii) redox-sensitive elements relevant to sediment–water exchange and metal cycling (Fe and Mn); and (iii) additional trace and major elements used to characterize watershed weathering, agricultural return-flow inputs, and ionic background conditions (Ni, Ba, Sb, Ca, and Mg).

### 2.7. Data Processing and Statistical Analysis

Data processing and statistical analyses were performed using MATLAB R2021a (MathWorks, Natick, MA, USA) and the DOMFluor toolbox for parallel factor analysis (PARAFAC) of the EEM datasets. Prior to PARAFAC modeling, all EEM spectra were corrected for instrumental biases and inner-filter effects, and normalized to Raman units based on the daily measured Raman scatter of ultrapure water [22,23]. Spectral indices, including fluorescence indices (FI, HIX, BIX) and ultraviolet-visible absorption indices (E2/E3, E2/E4, E4/E6) were calculated according to established methods as referenced in Section 2.4. For ion chromatography and ICP-MS data, instrument-specific software (Chromeleon 7.2 and MassHunter 4.5, respectively) was used for peak integration, baseline correction, and concentration quantification.

Statistical analyses were conducted using SPSS Statistics 26.0 (IBM, Armonk, NY, USA). Because DOM indices and total metal concentrations were both available for 17 co-located water stations, exploratory Spearman correlation analysis was used to examine associations within the water matrix only. This analysis was not used to infer metal–DOM complexation or causal regulation. All figures were prepared using OriginPro 2022 (OriginLab Corporation, Northampton, MA, USA), with consistent formatting applied across all graphical representations.

## 3. Results

### 3.1. Basic Water Quality and Pollution Characteristics of the Lake

#### 3.1.1. Analysis of Physicochemical Properties of the Lake Water

**Figure 2:**
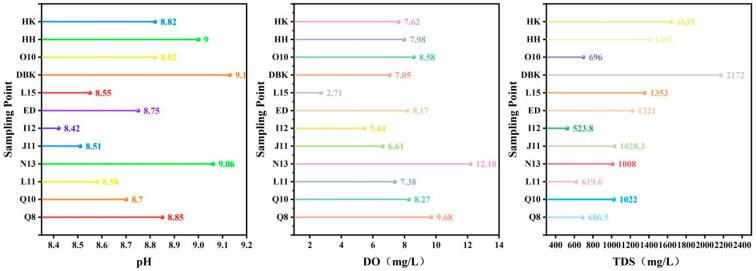
Water quality parameters of lake.

As shown in Figure 2, the water body of Ulansuhai Lake is weakly alkaline and exhibits high mineralization. The pH ranged from 8.42 to 9.13, with an average of 8.77. The dissolved oxygen (DO) concentration ranged from 2.71 to 12.2 mg/L, averaging 7.64 mg/L, with the lowest value recorded at site L15, indicating localized hypoxia in the lake area. Total dissolved solids (TDS) ranged from 524 to 2170 mg/L, with an average of 1030 mg/L, indicating elevated dissolved salt loads in parts of the lake. The highest TDS value was observed at the eastern shore of the lake and the lowest at the entrance to the lake, revealing significant spatial differences in water mineralization.

#### 3.1.2. Analysis of Spatial Distribution Characteristics of Anions and Cations in Multiple Media of the Lake

**Figure 3:**
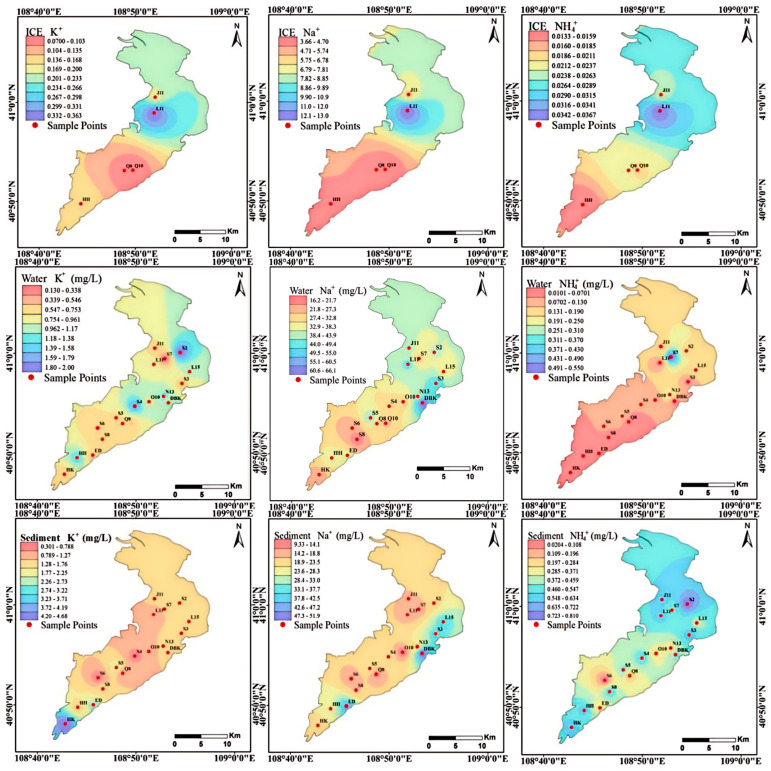
Distribution characteristics of anions and cations in ice, water, and sediment of the lake.

As shown in Figure 3, measured cation concentrations differed among analytical matrices. In water, mean Na^+^, NH_4_^+^, and K^+^ concentrations were 35.1, 0.090, and 0.890 mg/L, respectively, whereas the corresponding means in ice were 6.37, 0.020, and 0.170 mg/L. Sediment values (Na^+^: 23.9 mg/L; NH_4_^+^: 0.450 mg/L; K^+^: 1.53 mg/L) were determined in aqueous sediment extracts and are therefore reported as water-extractable fractions rather than directly compared with lake-water or ice concentrations.

The ion distribution results indicate that ice formation has a rejection and partitioning effect on solutes. The mean Na^+^ concentration was lower in ice (6.37 mg/L) than in lake water (35.1 mg/L), indicating that dissolved salts were preferentially retained in the liquid water phase during freezing. The sediment-extract value (23.9 mg/L) represents the water-extractable Na^+^ pool in sediment and is discussed as an operational extract fraction rather than as a directly comparable in situ water concentration.

**Figure 4:**
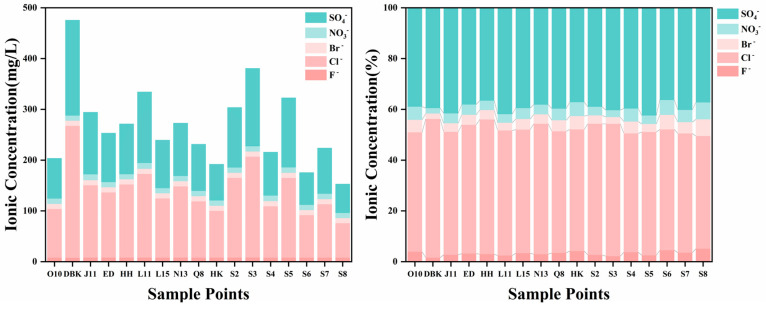
Distribution and percentage stacked chart of anion concentrations in lake water.

As shown in Figure 4, the anions in the water are dominated by Cl^−^ and SO_4_^2−^ (the sum of which accounts for 88.4% of the total anions), with average concentrations of 130 and 102 mg/L, respectively, and both reach their highest values at site DBK. The concentrations of F^−^, Br^−^, and NO_3_^−^ exhibit relatively small spatial differences, with average values of 7.53, 10.1, and 10.6 mg/L, respectively. The reported ionic composition is based on the measured anions and cations. The highest mineralization at DBK is attributed to the combined effects of salt-bearing agricultural return flow, shallow-lake evaporative concentration, and relatively weak hydrodynamic dilution near the eastern part of the lake; this interpretation is consistent with the simultaneous enrichment of TDS, Na^+^, Cl^−^, and SO_4_^2−^ at this site.

#### 3.1.3. Analysis of Spatial Distribution Characteristics of Trace Metals in Lake Water

**Figure 5:**
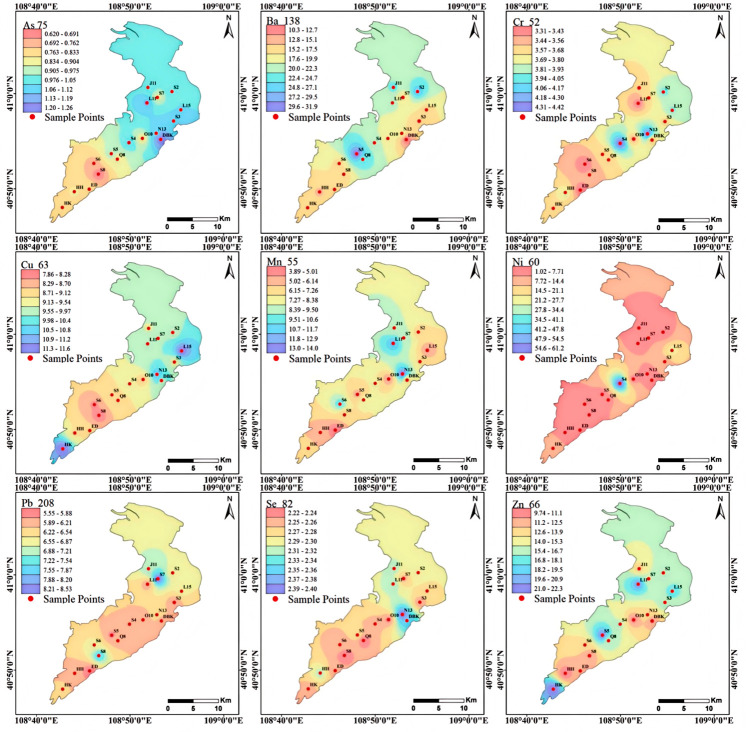
Spatial distribution of trace metal concentrations in lake water.

As shown in Figure 5, the ICP-MS results show that the concentrations of metals in the water body showed clear spatial variability. The concentrations of As, Ba, Cr, Cu, Mn, Ni, Pb, Se, and Zn ranged from 0.62 to 1.26, 10.3 to 31.9, 3.31 to 4.42, 7.86 to 11.7, 3.89 to 14.0, 1.02 to 61.2, 5.55 to 8.53, 2.22 to 2.40, and 9.73 to 22.3 μg/L, with average values of 0.94, 19.0, 3.68, 9.56, 7.48, 9.24, 6.41, 2.28, and 14.9 μg/L, respectively. Cd was reported as not detected in all water samples, with a method detection limit of 0.02 μg/L. Among the detected metals, Ni exhibited the most pronounced spatial variation, with the highest value observed at site S4 and the lowest at site S6. To evaluate water-metal contamination, the measured concentrations were compared with the Class III limits of the Environmental Quality Standards for Surface Water (GB 3838-2002), including As (50 μg/L), Cd (5 μg/L), Cr(VI) (50 μg/L), Cu (1000 μg/L), Pb (50 μg/L), Se (10 μg/L), and Zn (1000 μg/L). The measured As, Cd, Cu, Pb, Se, and Zn concentrations were below these criteria; Cr was measured as total Cr and was therefore interpreted together with spatial distribution rather than directly equated with Cr(VI). The observed metal distribution patterns are likely associated with agricultural return flow, sediment–water exchange under locally low DO, shallow-lake resuspension, and natural weathering inputs from the surrounding watershed.

### 3.2. Differences in Spectral Indices of DOM Components in Multiple Media of the Lake

The fluorescence indices and fluorescence components of dissolved organic matter (DOM) in the ice-water-sediment system of Ulansuhai Lake exhibit significant differences among media. The average fluorescence index (FI) of DOM in ice is 1.93, with the highest value (2.12) at site HHM, indicating a predominantly biological source of DOM in ice. The mean HIX of ice was 0.26, and the maximum HIX was 0.63 at L11D, indicating generally low humification relative to the other matrices. The mean BIX of ice was 1.23, with a maximum of 1.57 at Q8D, consistent with the presence of recently produced protein-like fluorescent DOM in the ice samples.

For water DOM, the mean FI was 1.94, with the maximum value of 2.26 at S4. The mean HIX was 1.88, with a maximum value of 2.38 at S7, indicating higher average humification than in ice and sediment-derived DOM. The mean BIX was 1.13, indicating a measurable contribution of recently produced fluorescent DOM.

For sediment-derived DOM, the mean FI was 1.95 and the maximum was 2.22 at S5. The mean HIX was 0.61, with a maximum of 0.92 at L11; sediment-derived DOM therefore showed higher average humification than ice but lower average humification than water. The mean BIX was 1.02. Because the material was obtained by water extraction of processed sediment, this BIX value may reflect water-extractable recent organic matter as well as extraction-dependent effects and should not be treated as evidence of in situ freshly produced sediment DOM.

**Figure 6:**
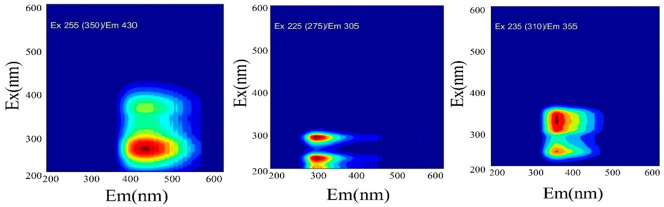
Fluorescence component spectra of DOM in lake ice, water, and sediment.

As shown in Figure 6, the PARAFAC results reveal that three fluorescent components are resolved in DOM from ice, water, and sediment, but the component types differ among media. Ice DOM consists of two humic-like components and one tyrosine-like protein component, corresponding to fulvic/humic acid-like substances (Ex/Em = 255[350]/430 nm), a humic-like component (Ex/Em = 235[310]/355 nm), and a tyrosine-like protein component (Ex/Em = 255[275]/305 nm). Water DOM includes a humic acid-like component (Ex/Em = 255[350]/430 nm), a tyrosine-like protein component (Ex/Em = 235[310]/355 nm), and a mixture of protein-bound amino acids (Ex/Em = 255[275]/305 nm). Sediment DOM mainly consists of a humic acid-like component, a tryptophan-like aromatic protein component, and an autochthonous organic matter-like humic component.

Overall, the mean HIX values followed the order water (1.88) > sediment-derived extract (0.61) > ice (0.26), while ice showed the highest mean BIX (1.23). These patterns identify matrix-specific differences in fluorescent DOM characteristics; because of non-contemporaneous sampling and operational sediment extraction, they do not demonstrate direct transformation from ice to water to sediment.

### 3.3. Humification Degree and Aromatic Structural Characteristics of DOM in Multiple Media of the Lake

**Figure 7:**
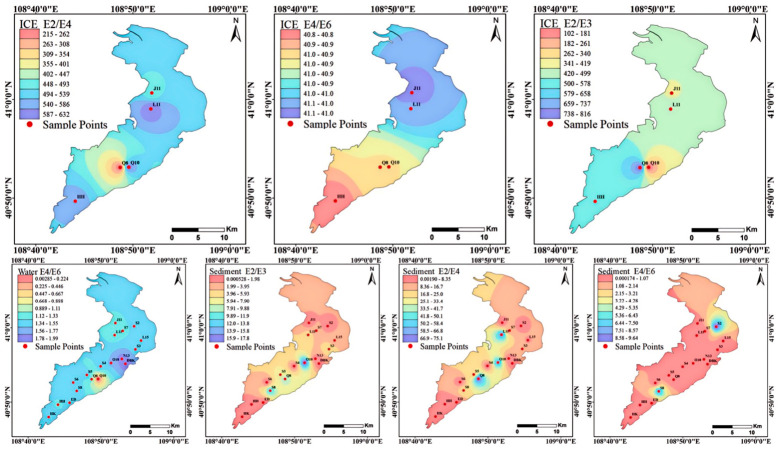
Spatial distribution of absorption index in multiple media of the lake.

As shown in Figure 7, the UV absorption indices of DOM in the ice-water-sediment system of Ulansuhai Lake exhibit differences among media. In ice samples, the E2/E3 and E2/E4 ratios at sites HHU, HHM, J11M, L11M and Q8M are relatively high. Because E2/E3 is inversely related to apparent molecular weight, high E2/E3 values are not interpreted as direct evidence of greater humification. The E4/E6 ratios are generally high across all ice sites, suggesting differences in conjugated and aromatic structural characteristics.

For water DOM, the E2/E3 and E2/E4 ratios are generally low. Except for site Q10, most water samples show high E4/E6 ratios. In sediment-derived DOM, sites L11, L15, O10, Q8, S3, S5, S7 and S8 exhibit measurable E2/E3 and E2/E4 ratios; site O10 has the highest E2/E3 ratio, and site Q8 has the highest E2/E4 ratio. The E4/E6 results show differences at sites S2 and S8. These UV-Vis ratios describe absorbance characteristics among matrices but do not independently establish a rank order of humification or metal-binding potential.

### 3.4. Analysis of Functional Group Structures of DOM Components in Lake Sediments

**Figure 8:**
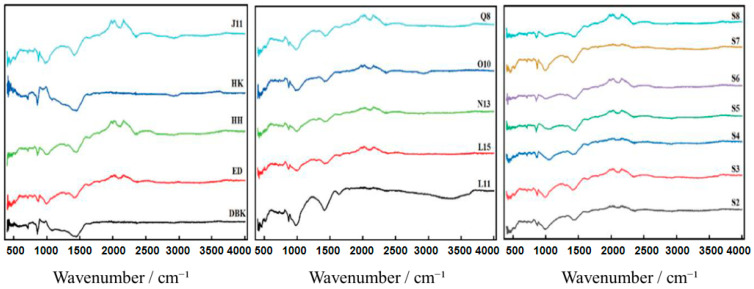
Functional group characteristic distribution of DOM components in lake sediments.

As shown in Figure 8, FTIR spectra of sediment DOM further reveal the presence of various organic functional groups. All samples exhibit an aromatic C=C stretching vibration peak in the range of 1620–1450 cm^−1^, and a C–O stretching vibration peak at approximately 1050 cm^−1^, corresponding to alcohols and polysaccharide structures. The absorption peak in the range of 740–680 cm^−1^ corresponds to benzene ring substitution C–H vibration. Except for sites DBK and HK, the remaining samples show an RC≡CH absorption peak in the range of 2100–2140 cm^−1^, indicating the presence of unsaturated organic structures. The absorption peak in the range of 1500–1800 cm^−1^ suggests the presence of peptide-like substances in sediment DOM. These structural characteristics indicate that sediment DOM contains both aromatic humic substances and biopolymers along with their degradation products.

## 4. Discussion

### 4.1. Associations Between DOM Characteristics and Measured Metal Distributions: Potential Mechanisms and Limitations

The composition, source, and molecular structure of dissolved organic matter (DOM) may influence the transport, transformation, and bioavailability of heavy metals in lakes [24,25,26,27,28]. In the present study, water DOM had the highest average HIX (1.88), followed by sediment-derived DOM (0.61) and ice DOM (0.26), whereas ice had the highest mean BIX (1.23). These patterns define differences in DOM spectral properties among analytical matrices.

Based on established knowledge, humic-like DOM typically contains abundant carboxyl, phenolic hydroxyl, and aromatic structures, which can enhance the binding capacity for metals or metalloids such as Cu, Pb, Cr, and As through complexation, chelation, and surface adsorption. In contrast, protein-like components are more representative of fresh autochthonous DOM; their nitrogen- and sulfur-containing functional groups may exhibit affinity for certain transition metals, but their high degradability may result in weaker binding stability of metal complexes [29,30]. UV-Vis and FTIR observations provide additional structural context. The UV absorption ratios indicate differences in absorbance characteristics among matrices, whereas FTIR confirms oxygen- and nitrogen-containing functional groups in sediment-derived DOM only. These spectral features suggest potential metal-binding environments.

**Figure 9:**
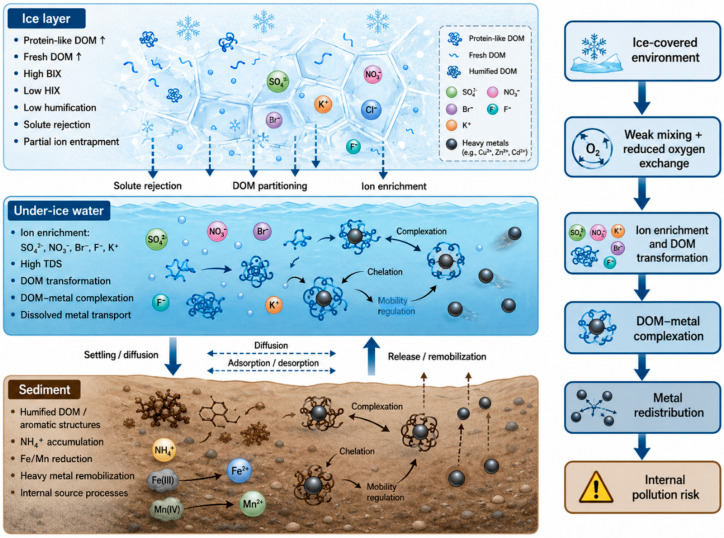
Mechanism diagram of the interaction between DOM components in lake ice, water and sediments and heavy metals. Arrows indicate the proposed directions of solute rejection, DOM partitioning, diffusion, release/remobilization, and DOM–metal interaction pathways.

However, direct measurements of metal–DOM complexation (e.g., through fluorescence quenching titration, equilibrium dialysis, or speciation modeling) were not performed in this study. Consequently, the associations between DOM characteristics and metal distributions remain speculative. This is further supported by the weak and statistically non-significant correlations observed for the 17 matched water stations, no Spearman correlation between FI, HIX, or BIX and an individual measured metal concentration reached *p* < 0.05; the strongest relationships were HIX-As (rho = 0.474, *p* = 0.054) and FI-Zn (rho = 0.471, *p* = 0.056). These results indicate that DOM optical properties alone cannot reliably predict metal concentrations in the water column under the conditions studied. Thus, the discussion above is intended to generate hypotheses about potential metal-interaction pathways rather than to establish causal relationships. Future studies incorporating direct metal-binding assays (e.g., complexation titrations) under site-specific conditions are needed to validate the proposed mechanisms. The near-threshold HIX-As and FI-Zn associations suggest that humification- and source-related DOM signals may be relevant to selected metals, but direct binding tests are required to confirm this mechanism, as conceptually summarized in Figure 9.

### 4.2. Potential Influence of Water Quality Conditions on DOM–Metal Associations

Water quality conditions may influence DOM–metal associations by altering metal speciation, DOM charge characteristics, and interfacial redox conditions [31,32]. The lake water is generally weakly alkaline, with pH ranging from 8.42 to 9.13. Weakly alkaline conditions can enhance the deprotonation of carboxyl and phenolic hydroxyl groups in DOM, increasing the negative charge density on DOM molecular surfaces, thereby enhancing their potential interactions with metal cations. At the same time, higher pH may also promote the occurrence of some metals in hydroxide, carbonate, or organic-associated forms. These pathways are chemically plausible, but they were not directly resolved by metal-speciation or binding analyses in the present study.

DO reflects the redox state of the water column and is an important factor controlling metal release and sediment–water interface exchange [33,34]. In this study, DO shows spatial differences, with the lowest value observed at site L15, indicating localized low-oxygen conditions (Figure 2). Under low-oxygen conditions, reductive dissolution of Fe/Mn oxides may release associated metals and DOM. Because this study did not measure sediment metal phases, pore-water fluxes, or metal speciation, L15 is identified as a priority site for future process measurements rather than as a demonstrated remobilization hotspot.

TDS represents dissolved salt load in the water column. In this study, the average TDS exceeds 1000 mg/L, with the highest TDS at site DBK (Figure 2). Higher ionic strength may affect DOM conformation, competition for binding sites, and inorganic metal complex formation. The measured pH, DO, and TDS therefore provide hydrochemical context for the metal distribution patterns, but they do not establish causal DOM–metal regulation.

### 4.3. Potential Influence of Major Ions on DOM–Metal Associations

Major cations and anions may influence DOM–metal associations through ionic-strength effects, competition, and inorganic complex formation [35,36,37]. The Na^+^ concentration measured in water is higher than that measured in ice, indicating a high salt load in the water column. Na^+^ itself typically does not form strong complexes with DOM, but it can alter DOM molecular conformation and colloidal stability as well as the accessibility of metal binding sites by increasing ionic strength. Sediment values represent water-extractable fractions and are not directly comparable to water or ice concentrations.

NH_4_^+^ and K^+^ were detected in aqueous sediment extracts, indicating extractable nutrient and cation pools under the applied extraction protocol. The accumulation of NH_4_^+^ is often associated with reducing environments and organic matter mineralization processes, and K^+^ may affect adsorption processes through ion exchange. However, because pore water and sediment metal-binding phases were not measured, the extract results cannot demonstrate in situ release, retention, or coupled transformation of metals at the sediment–water interface.

Cl^−^ and SO_4_^2−^ are the dominant measured anions in the water column and reach relatively high levels at site DBK. These anions can influence aqueous metal speciation in saline waters. However, bicarbonate, metal species, and binding equilibria were not determined; therefore, the measured ions define the ionic background rather than demonstrate specific complexation pathways.

### 4.4. Influence of Ice Cover Environment on DOM Properties and Heavy Metal Behavior

The ice cover environment provides unique physicochemical boundary conditions for DOM transformation and solute distribution in lakes. Compared with the open-water period, ice cover can reduce water-air exchange and wind-wave disturbance and can alter mixing and oxygen conditions. In the present dataset, ice DOM showed a high mean BIX and low mean HIX, while water showed the highest mean HIX. Because the samples were collected in different seasons, these contrasts are interpreted as matrix- and campaign-specific observations rather than direct winter transformation trajectories.

During ice formation, salts, DOM, and metals may undergo selective rejection, entrapment, or concentration. The lower measured Na^+^ concentration in ice than in water is consistent with possible solute exclusion, but contemporaneous paired ice-water data were not obtained. Therefore, quantitative partition coefficients for DOM or metals cannot be estimated from this dataset. Controlled freezing experiments and synchronized field sampling are needed to quantify partitioning.

Low temperature and weak disturbance during the ice-covered period may reduce microbial degradation rates and alter redox-sensitive exchange processes [38,39,40]. Local low-oxygen conditions can promote release of Fe/Mn-associated constituents from sediments, and released DOM may form soluble metal complexes [41,42]. In the present study, however, only total water-phase metal concentrations were measured; neither soluble DOM–metal complexes nor redox-mediated release processes were directly verified.

In summary, the DOM spectral patterns, measured water-quality variables, ion distributions, and exploratory correlation results in Lake Ulansuhai identify potential regulatory pathways for metal behavior under seasonal freezing. They do not prove direct in situ complexation or causal redistribution. However, humified DOM, labile protein-like DOM, weak alkalinity, high ionic strength, and ice-driven solute redistribution may jointly influence metal binding, speciation, and sediment–water exchange.

### 4.5. Limitations and Priorities for Further Research

This study does not provide direct measurements of metal–DOM complexation, metal speciation, DOC/TOC concentration, sediment total-metal inventories, sediment geochemical phases, or ice/liquid-water partition coefficients. Future studies should perform synchronized sampling of ice, under-ice water, sediment, and pore water; quantify DOC/TOC, alkalinity, sediment properties, and metal speciation; conduct direct binding experiments and geochemical modeling; and determine partition coefficients in controlled freezing experiments. Sediment contamination indices should be applied only when sediment metal concentrations and appropriate background values are available. These additional measurements will allow the potential DOM-related pathways identified here to be converted into quantitative metal–DOM binding and partitioning models. In particular, the absence of DOC/TOC data means that the optical indices should be interpreted as compositional indicators rather than concentration-normalized measures of DOM–metal binding capacity. Future sediment-focused assessments should also include sediment organic carbon, particle-size distribution, sediment pH, Fe/Mn oxide-bound phases, and bulk-sediment metal inventories so that enrichment factor (EF), geo-accumulation index (Igeo), and pollution load index (PLI) can be calculated with appropriate background values [43]. Following Tanaskovski et al. [43], these indices can be calculated as EF = (Cx/Fe)sample/(Cx/Fe)background, PLI = (CF_1_ × CF_2_ × … × CF_n_)^1^/^n^ Following Tanaskovski et al. [43], these indices can be calculated as follows: EF = (C_x_/Fe)sample/(C_x_/Fe)background, CF = Cmetal/Cbackground, Igeo = log_2_[Cn/(1.5Bn)], and PLI = (CF_1_ × CF_2_ × … × CF_n_)^1^/^n^, where CF is the contamination factor. with CF = Cmetal/Cbackground, and Igeo = log_2_[Cn/(1.5Bn)], providing a practical framework for interpreting the environmental significance of future sediment-metal datasets from Lake Ulansuhai.

## 5. Conclusions

This study reveals significant differences in the composition and sources of dissolved organic matter across the ice, water, and sediment media in Lake Ulansuhai. Based on the checked index table, water DOM showed the highest mean HIX (1.88), sediment-derived DOM had an intermediate mean HIX (0.61), and ice DOM had the lowest mean HIX (0.26). Ice DOM nevertheless showed the highest mean BIX (1.23), indicating a strong contribution of recently produced protein-like fluorescent material in the ice samples.

The results challenge the conventional view that “ice is a cleaner medium.” Although ice contained lower humification signals, its elevated BIX indicates that environmental interpretation should account for DOM composition rather than humification alone. The anomalous FI value at HHM requires verification before it is used for source attribution.

The measured DOM characteristics, ions, water-quality variables, and total metal concentrations identify potential associations relevant to winter monitoring, but they do not directly establish DOM-mediated metal complexation or redistribution. Further work should combine synchronized winter sampling, DOC/TOC and metal-speciation measurements, direct binding experiments, geochemical modeling, and controlled freezing tests before mechanistic conclusions or partition coefficients are established. The potential regulatory mechanisms involve humified DOM providing carboxyl, phenolic hydroxyl, and aromatic binding environments, protein-like DOM participating in short-term metal association, hydrochemical conditions modifying DOM charge and metal speciation, and ice cover promoting solute redistribution between ice and under-ice water.

## Data Availability

The raw data supporting the conclusions of this article will be made available by the authors on request.
